# Supplementary material for: Modeling glioblastoma heterogeneity as a dynamic network of cell states
Source: Mol Syst Biol. 2021 Sep 16;17(9):e10105. doi: 10.15252/msb.202010105 (PMC8444284; doi:10.15252/msb.202010105)
Supplement: Supplementary file 6 — Source Data for Figure 5 [file MSB-17-e10105-s004.zip › Figure5A_sourcedata/GSEA_3071/nosigdb_stateII.GseaPreranked.1622795151171/RICHARDS_NEFTEL_CELL_2019_NPC1.html]

Details for gene set RICHARDS\_NEFTEL\_CELL\_2019\_NPC1[GSEA]

|  || Dataset | state23071 |
| Phenotype | NoPhenotypeAvailable |
| Upregulated in class | na\_neg |
| GeneSet | RICHARDS\_NEFTEL\_CELL\_2019\_NPC1 |
| Enrichment Score (ES) | -0.75622284 |
| Normalized Enrichment Score (NES) | -3.199747 |
| Nominal p-value | 0.0 |
| FDR q-value | 0.0 |
| FWER p-Value | 0.0 |
Table: GSEA Results Summary

  

Fig 1: Enrichment plot: RICHARDS\_NEFTEL\_CELL\_2019\_NPC1      
 Profile of the Running ES Score & Positions of GeneSet Members on the Rank Ordered List

  

| PROBE | GENE SYMBOL | GENE\_TITLE | RANK IN GENE LIST | RANK METRIC SCORE | RUNNING ES | CORE ENRICHMENT || 1 | MEST |  |  | 563 | 0.593 | -0.1186 | No |
| 2 | STMN1 |  |  | 1249 | 0.387 | -0.2741 | No |
| 3 | PTPRS |  |  | 2253 | -0.254 | -0.5122 | No |
| 4 | PCBP4 |  |  | 2633 | -0.306 | -0.5951 | No |
| 5 | TSPAN13 |  |  | 2706 | -0.318 | -0.6023 | No |
| 6 | MLLT11 |  |  | 2760 | -0.328 | -0.6043 | No |
| 7 | HIP1 |  |  | 2814 | -0.338 | -0.6061 | No |
| 8 | ABAT |  |  | 3075 | -0.391 | -0.6569 | No |
| 9 | GLCCI1 |  |  | 3444 | -0.517 | -0.7302 | No |
| 10 | OLIG1 |  |  | 3551 | -0.571 | -0.7372 | Yes |
| 11 | CD24 |  |  | 3624 | -0.620 | -0.7342 | Yes |
| 12 | SERINC5 |  |  | 3676 | -0.664 | -0.7246 | Yes |
| 13 | TAGLN3 |  |  | 3751 | -0.729 | -0.7184 | Yes |
| 14 | SOX4 |  |  | 3797 | -0.786 | -0.7033 | Yes |
| 15 | PFN2 |  |  | 3841 | -0.832 | -0.6861 | Yes |
| 16 | MARCKSL1 |  |  | 3852 | -0.845 | -0.6604 | Yes |
| 17 | CHD7 |  |  | 3880 | -0.904 | -0.6368 | Yes |
| 18 | DLL3 |  |  | 3905 | -0.956 | -0.6108 | Yes |
| 19 | LRRN1 |  |  | 3913 | -0.970 | -0.5802 | Yes |
| 20 | BTG2 |  |  | 3917 | -0.999 | -0.5476 | Yes |
| 21 | TCF12 |  |  | 3931 | -1.022 | -0.5167 | Yes |
| 22 | ETV1 |  |  | 3934 | -1.026 | -0.4830 | Yes |
| 23 | HES6 |  |  | 3961 | -1.092 | -0.4530 | Yes |
| 24 | SOX11 |  |  | 3977 | -1.147 | -0.4184 | Yes |
| 25 | TUBA1A |  |  | 4006 | -1.302 | -0.3818 | Yes |
| 26 | TUBB3 |  |  | 4009 | -1.334 | -0.3378 | Yes |
| 27 | MAP2 |  |  | 4051 | -1.673 | -0.2921 | Yes |
| 28 | ASCL1 |  |  | 4061 | -1.762 | -0.2356 | Yes |
| 29 | BEX1 |  |  | 4072 | -2.034 | -0.1702 | Yes |
| 30 | FXYD6 |  |  | 4089 | -2.606 | -0.0871 | Yes |
| 31 | DCX |  |  | 4091 | -2.671 | 0.0017 | Yes |
Table: GSEA details [plain text format]

  

Fig 2: RICHARDS\_NEFTEL\_CELL\_2019\_NPC1: Random ES distribution      
 Gene set null distribution of ES for **RICHARDS\_NEFTEL\_CELL\_2019\_NPC1**
